# Isolation and Characterization of N-acyl Homoserine Lactone-Producing Bacteria From Cattle Rumen and Swine Intestines

**DOI:** 10.3389/fcimb.2018.00155

**Published:** 2018-05-09

**Authors:** Yang Yang, Mingxu Zhou, Philip R. Hardwidge, Hengmi Cui, Guoqiang Zhu

**Affiliations:** ^1^Institute of Epigenetics and Epigenomics and College of Animal Science and Technology, Yangzhou University, Yangzhou, China; ^2^Jiangsu Co-Innovation Center for Important Animal Infectious Diseases and Zoonoses, Yangzhou University, Yangzhou, China; ^3^College of Veterinary Medicine, Yangzhou University, Yangzhou, China; ^4^College of Veterinary Medicine, Kansas State University, Manhattan, KS, United States

**Keywords:** acyl-homoserine lactone, cattle rumen, *E. coli*, pig intestine, quorum sensing

## Abstract

Quorum sensing systems regulate gene expression in response to bacterial population density. Acyl-homoserine lactones are a class of quorum sensing molecules found in cattle rumen that are thought to regulate the gene expression of enterohemorrhagic *Escherichia coli* and thus help this pathogen survive in animal gastrointestinal tracts. However, the specific bacteria that produce these signaling molecules in bovine and porcine gastrointestinal tracts are unknown. Here we developed methods to concentrate gastrointestinal fluids and screen the bacteria that produce acyl-homoserine lactones. We isolated a *Pseudomonas aeruginosa* strain YZ1 from cattle rumen, and an *Aeromonas hydrophila* strain YZ2 from pig intestine. Mass spectrometry analysis of culture supernatants indicated at least three specific classes of acyl-homoserine lactones produced by YZ1, and a C4-acyl-homoserine lactone produced by YZ2. Transformation of *E. coli* with *P. aeruginosa* or *A. hydrophila luxI* homologs,which can produce short- or long-chain acyl-homoserine lactones conferred upon *E. coli* the ability to synthesize acyl-homoserine lactones and affected gene expression, motility, and acid tolerance of *E. coli*. This is the first study reporting the isolation and characterization of acyl-homoserine lactone synthase-positive bacteria from cattle rumen and swine intestines.

## Introduction

Quorum sensing (QS) systems regulate gene expression in response to bacterial population density. Small molecules named autoinducers (AIs) are produced, released, and detected in the QS process. The LuxI/LuxR QS-I system was first described in the bioluminescent marine bacterium *Vibrio fischeri*. When QS-I threshold concentrations are reached, diverse biological processes are affected, including symbiosis, virulence, competence, conjugation, antibiotic production, motility, sporulation, and biofilm formation (Reading and Sperandio, 2006; Walters and Sperandio, 2006; Waters et al., 2008; Boyen et al., 2009).

Cattle are the primary animal reservoir of enterohemorrhagic *E. coli* (EHEC) (Andrade et al., 2012; Borriello et al., 2012; Duan et al., 2012, 2013; Yang et al., 2013), an important foodborne pathogen that causes outbreaks of hemorrhagic colitis and hemolytic uremic syndrome (Barlow and Mellor, 2010; Sperandio, 2010). Shiga-like toxin producing (SLTEC) and verotoxigenic *E. coli* (VTEC) cause both porcine edema disease (ED) and post-weaning diarrhea (PWD) (da Silva et al., 2001; Frydendahl, 2002; Mainil et al., 2002). *E. coli* encodes a single LuxR homolog named SdiA, but is believed not to express the LuxI homolog, the acyl-homoserine lactone (AHL) synthase, which produces AI-1 (Ahmer, 2004; Yao et al., 2006; Smith et al., 2011). In the presence of exogenous AHLs, a significant proportion of SdiA, the AHL receptor, is expressed in a folded, soluble form, while it is expressed in insoluble inclusion bodies in the absence of AI-1. Thus, *E. coli* can regulate its virulence gene expression in response to the population density of other AHL-positive bacteria (Dyszel et al., 2010; Soares and Ahmer, 2011; Yakhnin et al., 2011). These responses include cell division (Yamamoto et al., 2001; Shimada et al., 2014), antibiotic resistance (on plasmid-encoded *sdiA* condition) (Rahmati et al., 2002; Van Houdt et al., 2006; Dyszel et al., 2010; Lu et al., 2017; Zhang et al., 2018), motility (Kanamaru et al., 2000; Wei et al., 2001; Yang et al., 2013), and the activity of other QS systems (Yang et al., 2013).

Different AHLs (C6-HSL, C8-HSL) have been detected in cattle rumens (Erickson et al., 2002; Hughes et al., 2010). QS signals in the rumen repress the expression of the locus of enterocyte effacement (*LEE*) genes and activate the expression of the glutamate decarboxylase A (*gadA*) gene in EHEC, suggesting QS-I signals modulate EHEC passage through the bovine gastrointestinal tract (Dyszel et al., 2010; Sperandio, 2010; Sheng et al., 2013; Nguyen et al., 2015), perhaps influencing EHEC survival in acidic environments. However, the specific source of these QS-1 signals is currently lacking, as, to our knowledge, there are no reports of the isolation and identification of AHL-producing bacteria from cattle rumen (Erickson et al., 2002; Soares and Ahmer, 2011).

AHL activities *in vivo* have been examined previously from the gastrointestinal tracts of various animals, including pigs, using a reporter strain method (Smith et al., 2008). The lack of evidence for AHL-producing bacteria in pig intestines has also hindered in-depth studies of QS-I function on *E. coli* in pigs. The QS-II system has been studied in porcine (Zhu et al., 2011; Yang et al., 2014), bovine (Sperandio et al., 2002), and avian (Palaniyandi et al., 2013) pathogenic *E. coli*, and has been shown to affect virulence gene expression. Here we developed a method with which to concentrate gastrointestinal tract samples and screen the contents for AHL–producing bacteria. We isolated and characterized an N-acyl homoserine lactone-producing *Pseudomonas aeruginosa* strain from cattle and an *Aeromonas hydrophila* strain from pigs. We also determined the extent to which QS-I signals from these strains impact *E. coli* virulence gene expression *in vitro*.

## Materials and methods

### Bacterial strains and growth conditions

The bacterial strains and plasmids used are listed in Table 1. *E. coli* 107/86 (wild-type, O139:H1: F18ab, Stx2e) (Bertschinger et al., 1990) was cultured in Luria broth (LB) or on Luria agar (LA) plates at 37°C. *A. hydrophila* J-1 (Yan et al., 1996) was used as positive control for *ahyI* gene expression. *Yersinia enterocolitica* GIM1.266 was used as a positive control in AHL cross-streaking assays. *E. coli* DH5α was used as a negative control. *Agrobacterium tumefaciens* JZA1 (Fuqua and Winans, 1996) and *Chromobacterium violaceum* CV026 (Latifi et al., 1995) were cultured in Luria broth (LB) or on Luria agar (LA) plates at 30°C. The bioluminescence reporter strains *E. coli* pSB401 and pSB1142 (Winson et al., 1998) were used as short- and long-side chain AHLs biosensors, respectively, and were grown in LB at 37°C.

**Table 1:** Strains and plasmids used in this study.

**Strain/plasmid**	**Description**	**Source/references**
**STRAINS**		
*A. tumefaciens* JZA1	AHL biosensor	Fuqua and Winans, 1996
*C. violaceum* CV026	AHL biosensor	Latifi et al., 1995
*Escherichia coli* pSB401	AHL biosensor	Winson et al., 1998
*E. coli* pSB1142	AHL biosensor	Winson et al., 1998
*E. coli* O157:H7 8624	Wild-type EHEC	Havens et al., 1992
*E. coli* 107/86	Wild-type STEC	Bertschinger et al., 1990
*E. coli* 8624/pBR322	*E. coli* 8624 carrying pBR322	This study
*E. coli* 8624/p27853-*lasI*	*E. coli* 8624 carrying p27853-*lasI*	This study
*E. coli* 8624/p27853-*rhlI*	*E. coli* 8624 carrying p27853-*rhlI*	This study
*E. coli* 8624/pYZ1-*lasI*	*E. coli* 8624 carrying pYZ1-*lasI*	This study
*E. coli* 8624/pYZ1-*rhlI*	*E. coli* 8624 carrying pYZ1-*rhlI*	This study
*E. coli* 107/86/pBR322	*E. coli* 107/86 carrying pBR322	This study
*E. coli* 107/86/pJ1-*ahyI*	*E. coli* 107/86 carrying pJ1-*ahyI*	This study
*E. coli* 107/86/pYZ2-*ahyI*	*E. coli* 107/86 carrying pYZ2-*ahyI*	This study
*Pseudomonas aeruginosa* ATCC27853	*lasI/rhlI* cloning	D'Amato et al., 1975
*P. aeruginosa* YZ1	Strain isolated from cattle rumen	This study
*A. hydrophila* J-1	*ahyI* cloning	Yan et al., 1996
*A. hydrophila* YZ2	Strain isolated from pig intestines	This study
*Yersinia enterocolitica* GIM1.266	AI-1 bioassay positive control	Guangdong Inst. Microbiology
**PLASMIDS**		
pBR322	Expression vector, Amp^r^	Takara Ltd.
pJ1-ahyI	J-1 *ahyI* in pBR322	This study
pYZ2-ahyI	YZ2 *ahyI* in pBR322	This study
p27853-*lasI*	ATCC27853 *lasI* in pBR322	This study
p27853-*rhlI*	ATCC27853 *rhlI* in pBR322	This study
pYZ1-*lasI*	YZ1 *lasI* in pBR322	This study
pYZ1-*rhlI*	YZ1 *rhlI* in pBR322	This study

### Isolation of AHL-producing bacteria

Cattle rumen fluids from cattle rumen fistula and pig intestinal samples were obtained from the Animal Hospital of Yangzhou University according to the Guide for the Care and Use of Laboratory Animals of the Ministry of Science and Technology of the People's Republic of China. The protocols for animal experiments were approved by the Jiangsu Administrative Committee for Laboratory Animals (approval number: SYXK-SU-2007-0005), and complied with the guidelines of Jiangsu laboratory animal welfare and ethics of Jiangsu Administrative Committee of Laboratory Animals. Rumen contents were filtered through six layers of hospital gauze. The filtered rumen fluid and PBS-washed intestinal samples were centrifuged at 10,000 ^*^g for 10 min, and the pellets were spread on LB plates and incubated at 37°C for 24 h. Isolated bacterial colonies were inoculated into 8 ml LB for overnight growth, after which the culture supernatants were extracted in 2 ml ethyl acetate (Sperandio, 2010). The organic phase was collected and evaporated to dryness. Twenty microliters of Milli-Q water was added to dissolve the dried contents, then seeded onto a 0.8% LA plates spread with both X-gal (50 μg/ml) and the reporter strain JZA1 (5 × 10^7^ CFU/ml).

### Cross-streaking assay

Cross-streaking assays were performed as described previously (Yang et al., 2013). The reporter strain CV026 was spread in the middle of an LA plate, and tested strains were streaked perpendicular to CV026. After overnight growth, purple color induced in CV026 was observed visually. Similarly, a JZAI-based cross-streaking assay for long-side chain AHLs detection was conducted.

### Strain phylogenetic analysis

The partial 16S rDNA genes from YZ1 and YZ2 were amplified using PCR (Chen et al., 2013) with primers 16SF and 16SR (Table 2) and subsequently sequenced. Related nucleotide sequences available in GenBank were aligned and phylogenetic analysis was conducted using MEGA 4.0.

**Table 2:** Primers used in this study.

**Primer**	**Sequence (5′-3′)**
16SF	AGAGTTTGATCCTGGCTCAG
16SR	GGCTACCTTGTTACGACT
*rhlI*-F	CGCGGATCCATGATCGAATTGCT
*rhlI*-R	TTAGTCGACTCACACCGCCATCG
*lasI*-F	CGCGGATCCATGATCGTACAAATT
*lasI*-R	TTAGTCGACTCATGAAACCGCCAGT
*ahyI-F*	CGAGCTAGCATGCTTGTTTTCAAAG
*ahyI-R*	TAAGTCGACTTATTCGGTGACCAGT
*fliC-*EHEC-RT-F	TGTGACTGTTGCCGGGTATG
*fliC*-EHEC-RT-R	AGTGATTTTACCCGCGGAGTT
*fliC-*STEC-*RT-F*	CAGCAAGCGGTGAAGTGAA
*fliC-*STEC-*RT-R*	AAGCGTAGCCACAGTAGCA
*sdiA*-RT-F	GCGTCGCACGATGCTGTT
*sdiA*-RT-R	CCCACGCCTCAGGGTAAT
*gadA*-RT-F	AGGCAAACCAACGGATAAACC
*gadA*-RT-R	GGCGCATAGGGATCTCACG
*gapA*-RT-F	CGTTAAAGGCGCTAACTTCG
*gapA*-RT-R	ACGGTGGTCATCAGACCTTC

### AHL extraction

To obtain sufficient AHLs for biochemical characterization, YZ1 and YZ2 were inoculated into 50 ml LB respectively and, from 2 to 12 h growth, were extracted vigorously with ethyl acetate as described (Chen et al., 2013). The organic phases were collected and filtered through a 0.22 μm filter membrane, evaporated, and dissolved in 100 μl of Milli-Q water.

### β-Galactosidase assays

An overnight culture of JZA1 (2 × 10^9^ CFU/ml) was diluted 1:100 into 1 ml fresh LB medium and 100 μl extracts of bacterial cultures or rumen fluids were added. After 12 h incubation, β-galactosidase activity for each sample was determined using ONPG as described (Tran et al., 2001).

### Bioluminescence assays

The *lux*-based biosensor *E. coli* strains pSB401 and pSB1142 were used to determine inducible *lux* bioluminescence activity activated by short- and long-side chain AHLs, respectively (Winson et al., 1998). Overnight cultures of *E. coli* pSB401 and pSB1142 were diluted 1:100 into 1 ml fresh LB medium and 100 μl extracts of bacterial cultures or rumen fluids were added. After 6 h incubation, bioluminescence was measured using a Tecan GENios Plus microplate reader in luminescence mode (TECAN GmbH, Austria). Data were expressed as relative light units of luminescence values from each sample.

### Mass spectrometry

High performance liquid chromatography mass spectrometry was used to analyze AHL production by YZ1 and YZ2 using a LCQ Deca XP max (Thermo Finnigan, USA; Yin et al., 2012; Chen et al., 2013). Liquid chromatography steps utilized a C18 column of 4.6 × 150 mm, 5 μm particle size, with injection volume of 20 μl and a flow rate of 0.3 ml/min. Mobile phases were 0.1% *v*/*v* formic acid in water and 0.1% *v*/*v* formic acid in acetonitrile, respectively. Mass spectrometry was run in ESI-positive mode, with probe capillary voltage set at 3,000 V, a desolvation temperature of 350°C, sheath gas of 11 ml/h, and a nebulizer pressure of 50 psi (Chen et al., 2013).

### Expression and analysis of *luxI* homologous genes in *E. coli*

The *luxI* homologs, *rhlI* and *lasI* from YZ1 and *P. aeruginosa* ATCC27853 were PCR-amplified by using two pairs of specific primers (designed according to Accession No. CP006728.1). PCR products were ligated into pBR322 and transformed into *E. coli* O157:H7 strain 8624. The *ahyI* genes from YZ2 and *A. hydrophila* J-1 were transformed into *E. coli* F18 107/86 (primers were designed according to Accession No. X89469.1). Total RNA from recombinant *E. coli* strains were extracted using TRIzol as described previously (Yang et al., 2013). Data were normalized to the endogenous reference gene *gapA* and analyzed using the 2^ΔΔCT^ method. For motility assays, *E. coli* strains were seeded in the middle of motility plates and motility halos were subsequently measured. For acid tolerance assays, *E. coli* strains were seeded in acidified LB (pH 2.5) supplemented with 1.0 mM glutamate (Dyszel et al., 2010). Bacterial survival was enumerated from 0 to 2 h.

### Statistics

Quantitative data are shown as the mean ± standard error of at least three independent experiments. Data were analyzed using two-tailed *t*-tests with asterisks indicating statistical significance (*p* < 0.05).

## Results

### Isolation of AHL-producing bacteria

**Figure 1:**
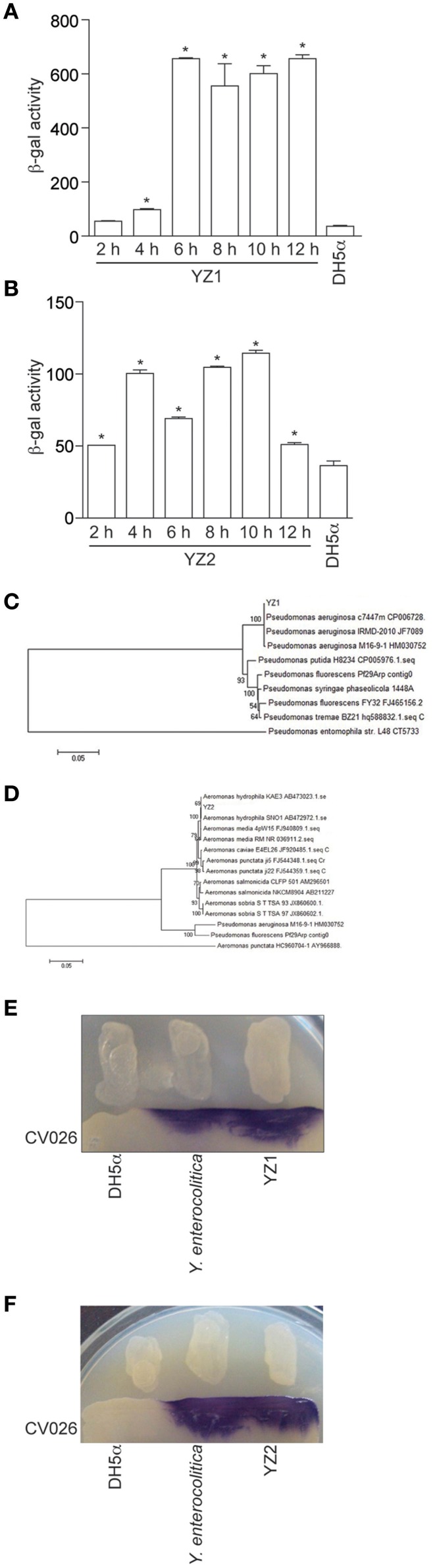
Isolation of the AHL-producing bacteria YZ1 and YZ2. **(A,B)** JZA1-based β-galactosidase assays of AHL production. β-galactosidase activity is plotted as a function of the culture supernatant added to JZA1 cultures. Strains (YZ1, **A**; YZ2, **B**) were grown for different times before the addition of supernatants, as indicated. Asterisks indicate significantly different β-galactosidase activity as compared with activity induced by DH5α supernatant. **(C,D)** Phylogenetic analyses of YZ1 and YZ2. The bar represents evolutionary distance as number of changes per nucleotide position, determined by measuring the lengths of the horizontal lines connecting the corresponding species. GenBank accession numbers are indicated. **(E,F)** Biosensor strain *C. violaceum* CV026-based cross-streaking assays for AI-1 activity (purple color). *P. aeruginosa* strains YZ1 **(E)** *A. hydrophila* YZ2 **(F)** the positive control *Y. enterocolitica* GIM1.266, and the negative control DH5α were struck across the biosensor strain as indicated.

To screen cattle rumen fluid and pig intestinal samples for bacteria that produce AHLs, we filtered rumen fluids and scraped pig intestinal walls to collect samples, then inoculated LB plates with the filtered or PBS-washed contents. In each case, we screened ~500 isolated bacterial colonies for AHL production by seeding the individual colonies into LB for overnight growth and then extracted culture supernatants with ethyl acetate. These extracts were added to the AHL reporter strain JZA1 in the presence of X-gal to monitor AHLs in the extracted supernatants by quantifying AHL-dependent JZA1 β-galactosidase activity. We found that the culture supernatant derived from two single isolated colonies (here designated as YZ1 from cattle rumen fluid and YZ2 from pig intestinal contents) induced β-galactosidase activity (Figures 1A,B). β-galactosidase activity was highest when YZ1 was grown for at least 6 h (Figure 1A) and was more variable for YZ2 (Figure 1B).

To begin to characterize the isolated strains, the partial 16S rDNA (1,528 bp) and AHL synthase genes (606 bp *lasI/rhlI* gene in YZ1 and 624 bp *ahyI* gene in YZ2) were sequenced. The sequencing data indicated strong homology (99%) between *Pseudomonas aeruginosa* ATCC27853 and YZ1 (Figure 1C), and strong homology (99%) between *A. hydrophila* J-1 and YZ2 (Figure 1D). Additionally, the colony odor and morphology of YZ1 and YZ2, oxidase production, and their ability to metabolize glucose, lactose, and sucrose were similar to that of other *P. aeruginosa* or *A. hydrophila* strains (data not shown). Hence, strain YZ1 was subsequently designated as *P. aeruginosa* YZ1, and YZ2 as *A. hydrophila* YZ2.

Because we initially used the JZA1 reporter assay to detect AHL-producing bacteria, we next sought to confirm these data using an independent method to avoid false positive results. We therefore utilized the biosensor strain *C. violaceum* CV026 in cross-streaking assays. Cross-streaking of YZ1 (Figure 1E) and YZ2 (Figure 1F), as well as the positive control strain *Yersinia enterocolitica* GIM1.266 on *C. violaceum* CV026 induced the formation of purple colonies, indicating that both YZ1 and YZ2 secrete AHLs.

**Figure 2:**
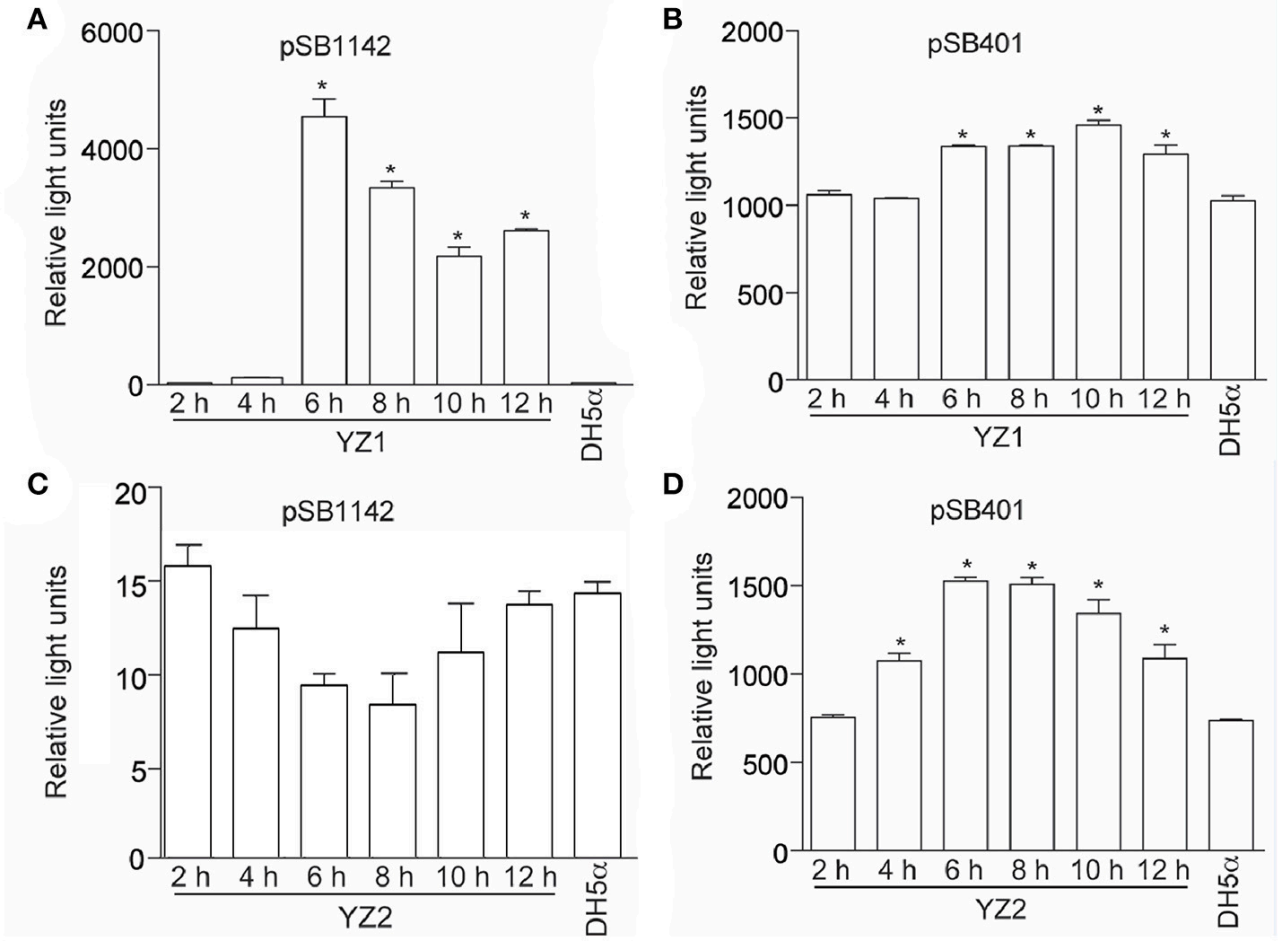
Bioluminescence assays of AHL production. **(A)** Bioluminescence (relative light units) of reporter strain *E. coli* pSB1142 after the addition of the indicated culture supernatants from YZ1. **(B)** Bioluminescence (relative light units) of reporter strain *E. coli* pSB401 after the addition of the indicated culture supernatants from YZ1. **(C)** Bioluminescence (relative light units) of reporter strain *E. coli* pSB1142 after the addition of the indicated culture supernatants from YZ2. **(D)** Bioluminescence (relative light units) of reporter strain *E. coli* pSB401 after the addition of the indicated culture supernatants from YZ2. Asterisks indicate significantly different reporter strain bioluminescence as compared with activity induced by *DH5*α supernatant.

To differentiate between short- and long-side chain AHL productions, we next employed two different *E. coli* biosensor strains. Both *E.coli*/pSB401 and *E. coli*/pSB1142 contain the luxCDABE cassette. *E.coli*/pSB401 emits light in the presence of short chain AHLs, while *E. coli*/pSB1142 is activated by long chain AHLs. The addition of YZ1 extracts to *E. coli*/pSB1142 induced bioluminescence of this reporter strain, indicating that YZ1 synthesized long chain AHLs (Figure 2A). YZ1 extracts also activated *E.coli*/pSB401 bioluminescence to an extent greater than that of the negative control strain, *E. coli* DH5α (Figure 2B). YZ2 extracts could only induce *E.coli*/pSB401 bioluminescence (Figures 2C,D). We therefore assumed that YZ1 secretes both short- and long-chain AHLs and YZ2 only secretes short-side chain AHLs.

### MS analysis of YZ1/YZ2 AHLs

**Figure 3:**
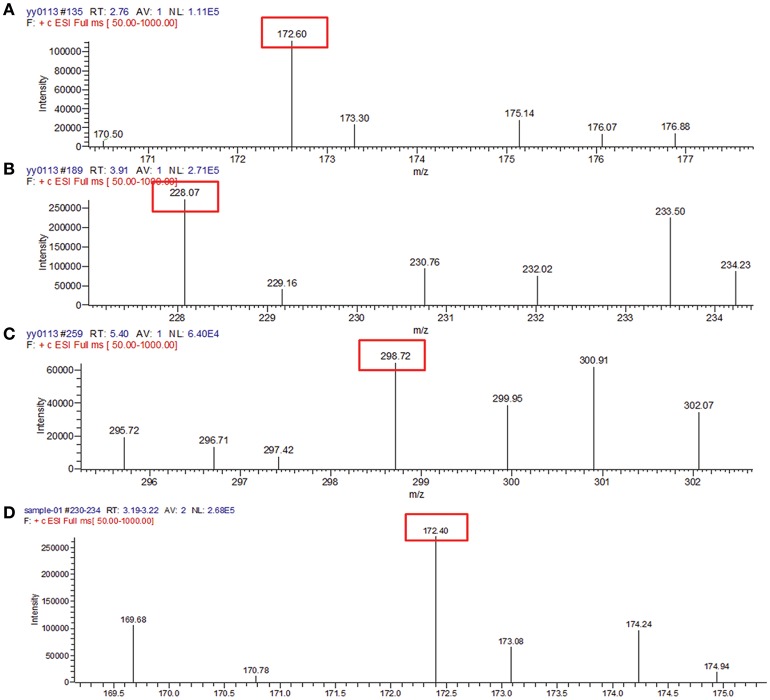
Mass spectrometry analysis of AHLs in YZ1 and YZ2 extracts**. (A)** Identification of C4-HSL produced by YZ1. **(B)** Identification of C8-HSL produced by YZ1. **(C)** Identification of 3-oxo-C12-HSL produced by YZ1. **(D)** Identification of C4-HSL produced by YZ2.

To identify the AHLs produced by YZ1 and YZ2, we employed mass spectrometry. More than 30 types of AHL signals have been identified in various bacterial QS systems (Gould et al., 2006). Previous papers have summarized the specific m/z data of ions from each AHL, which provided us sufficient information with which to identify the specific AHL produced by YZ1 and YZ2 (Ortori et al., 2007). By analyzing the contents of concentrated supernatants, in YZ1 we detected very similar mass peaks (Figures 3A–C) compared with C4-AHL (m/z 172.60), C8-AHL (m/z 228.07), and 3-oxo-C12-AHL (m/z 298.72), and in YZ2 we detected similar mass peaks (Figure 3D) compared with C4-AHL(m/z 172.40).

### Expression of *luxI* homologous genes in *E. coli*

*P. aeruginosa* possesses two AHL-dependent QS circuits, *rhlRI* and *lasRI* systems, which are responsible for the production of short- and long-chain AHLs, respectively (Chen et al., 2013). The *luxI* homologs *rhlI* and *lasI* from both *P. aeruginosa* YZ1 and ATCC27853 were PCR-amplified and cloned into pBR322. The recombinant plasmids were transformed into *E. coli* O157:H7 strain 8624, to determine whether endogenous expression of these genes would confer upon *E. coli* the ability to express AHLs. The same method was also used to study the *ahyI* gene from *A. hydrophila*, which was transformed into F18 *E. coli* 107/86.

**Figure 4:**
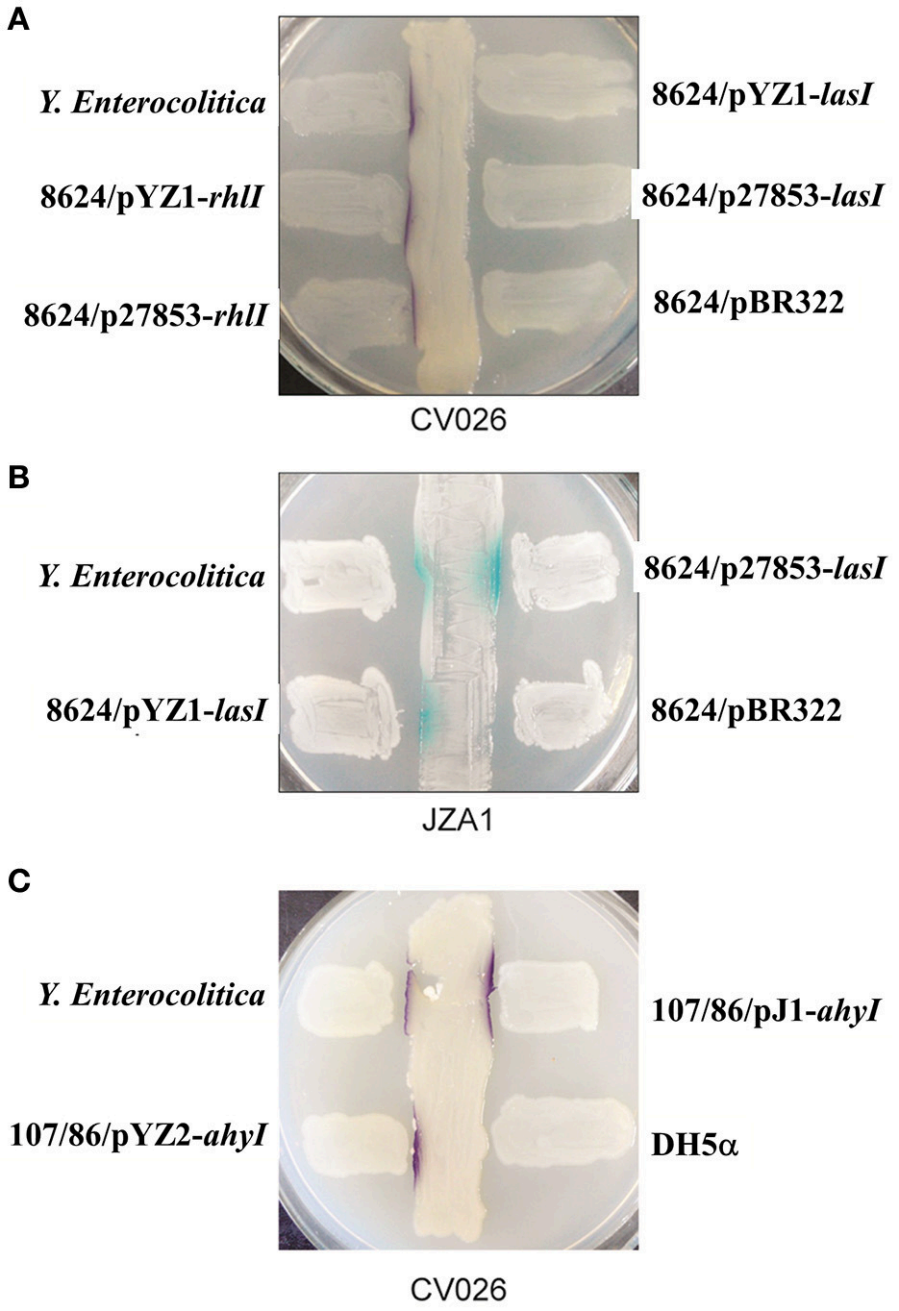
Cross-streaking assays for AI-1 activity from *E. coli* 8624 transformed with *lasI* or *rhlI* and *E. coli* 107/86 transformed with *ahyI*. **(A)** Biosensor strain *C. violaceum* CV026-based cross-streaking assay. The indicated *E. coli* 8624 recombinant strains were struck across the CV026 biosensor strain to detect short-chain AHLs (purple color). **(B)** Biosensor strain JZA1 assay. The indicated *E. coli* 8624 recombinant strains were struck across the JZA1 biosensor strain to detect long-chain AHLs (blue color). **(C)** CV026-based cross-streaking assay. The indicated *E. coli* 107/86 recombinant strain was struck across the CV026 biosensor strain to detect short-chain AHLs.

Cross-streaking of *E. coli* 8624 expressing *rhlI* from both *P. aeruginosa* YZ1 and ATCC27853 on *C. violaceum* CV026 induced the formation of purple colonies, suggesting that expressing *rhlI* in *E. coli* conferred the expression of short chain AHLs (Figure 4A; Pearson et al., 1995). We subsequently used the JZA1 reporter assay to quantify long-chain AHL-dependent β-galactosidase activity. These cross-streaking experiments also induced β-galactosidase activity, suggesting long chain AHL production in *E. coli* after transformation with *lasI* (Figure 4B). Cross-streaking of *E. coli*107/86 expressing *ahyI* from both *A. hydrophila* J-1 and YZ2 on CV026 induced the formation of purple colonies, suggesting that expressing *ahyI* conferred the expression of short chain AHLs (Figure 4C).

**Figure 5:**
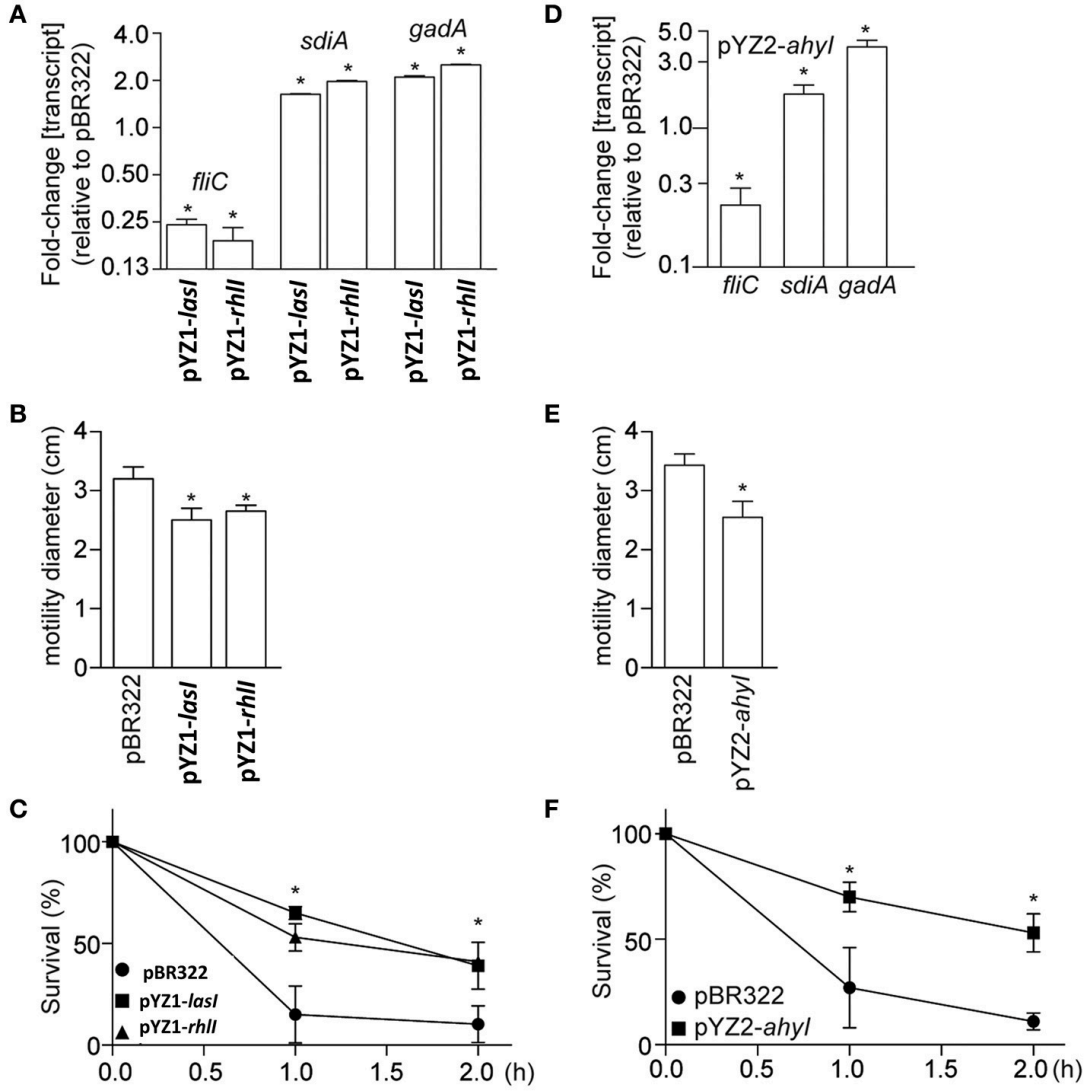
Phenotypic analysis of *E. coli* 8624 and 107/86 recombinant strains. **(A)** Relative *fliC, sdiA*, and *gadA* transcript abundance (as compared with *E. coli* 8624 transformed with pBR322) are plotted as a function of *E. coli* transformation with *luxI* homologs. Asterisks indicate significantly different gene expression, as compared with *E. coli* 8624 transformed with pBR322. **(B)** Motility diameters were measured after 12 h growth of the indicated strains on 0.3% swim agar plates. Asterisks indicate significantly different motility halos, as compared with *E. coli* 8624 transformed with pBR322. **(C)** Acid tolerance was quantified by enumerating bacteria survival as a function of time after growth seeded in acidified LB (pH 2.5) supplemented with 1.0 mM glutamate. Asterisks indicate significantly different survival, as compared with *E. coli* 8624 transformed with pBR322. **(D)** Relative *fliC, sdiA*, and *gadA* transcript abundance (as compared with *E. coli* 107/86 transformed with pBR322) are plotted as a function of *E. coli* transformation with *ahyI* from YZ2. Asterisks indicate significantly different gene expression, as compared with *E. coli* 107/86 transformed with pBR322. **(E)** Motility diameters were measured after 12 h growth of the indicated strains on 0.3% swim agar plates. Asterisks indicate significantly different motility halos, as compared with *E. coli* 107/86 transformed with pBR322. **(F)** Acid tolerance was quantified by enumerating bacteria survival as a function of time after growth seeded in acidified LB (pH 2.5) supplemented with 1.0 mM glutamate. Asterisks indicate significantly different survival, as compared with *E. coli* 107/86 transformed with pBR322.

Expressing the *luxI* homologs *lasI* and *rhlI* in *E. coli* affected the expression of several genes known to be regulated by AHLs, including *fliC, gadA*, and *sdiA* (Figure 5A). Consistent with these gene expression data, *E. coli* motility was inhibited (Figure 5B), whereas acid tolerance was enhanced (Figure 5C). Similar to the data obtained using YZ1, we also found that expressing ahyI from YZ2 in *E. coli* affected *E. coli* virulence gene expression, motility, and acid resistance (Figures 5D–F).

## Discussion

The identification of QS signaling molecules and the bacterial strains that produce these molecules is of considerable significance (Sperandio, 2010; Soares and Ahmer, 2011). A variety of bioassay strains have been developed to facilitate the detection of AHLs. Ideally, such a strain would contain an easily assayable reporter construct, lack endogenous AHL synthases, and be able to respond to exogenous AHLs (Zhu et al., 2003). However, most bioassay strains detect only a subset of AHLs. *A. tumefaciens* JZA1 is considered to be perhaps the most sensitive bioassay strain and can detect a wide range of AHLs (Zhu et al., 2003). However, it had not, to our knowledge, previously been utilized successfully to screen AHL-producing bacteria in cattle rumen fluids or pig intestinal contents (Erickson et al., 2002; Smith et al., 2008).

We therefore introduced enrichment, extraction, and evaporation steps to concentrate culture supernatants derived from rumen fluids or intestinal contents. By employing these modifications, we identified the AHL-producing bacterium *P. aeruginosa* YZ1 in cattle rumen, and *A. hydrophila* YZ2 in pig intestine. *A. hydrophila* was previously characterized for its quorum sensing functions upon *Salmonella enterica* in the gastrointestinal tract of turtles (Smith et al., 2008). Mass spectrometry analysis indicated the production of AHLs by YZ1 and YZ2. By cloning *luxI* homologs from YZ1 or YZ2, we conferred upon *E. coli* the ability to synthesize AHLs, to examine whether AHLs from specific environment could induce QS related pathogenic gene in *E. coli*.

*E. coli* inhabit the intestinal environment, and play important roles in humans and many other animals. Because its lack in AHL production, the simplest hypothesis is that in intestinal environment *E. coli* use SdiA to detect exogenous AHL, through which its pathogenicity could be regulated by these AHL-positive strains. However, this hypothesis appears to be incorrect, as chemical extractions and a *Salmonella* SdiA reporter both failed to detect AHLs within mammalian intestines (Soares and Ahmer, 2011; Swearingen et al., 2012). The failure of screening for AHL-positive bacteria in the digestive tract has directly affected the in-depth study for the synergistic regulation mechanism in pathogenicity of the intestinal bacteria, especially *E. coli*. Although this paper failed to answer whether YZ1 and YZ2 could synthesize AHL in the gut environment or synthesize sufficient AHL to activate QS-1 system in *E. coli*, it still emphasize the possibility that *E. coli* in relative environments could be regulated by AHL signals from other AHL-positive strains.

Biosensors have detected acyl-HSLs in chemical extracts of cow rumens, and rumen AHLs are known to repress expression of the *E. coli* LEE and activate *gadA* expression to improve acid tolerance (Dyszel et al., 2010; Sperandio, 2010; Sheng et al., 2013), while pig intestinal AHLs still have unclear functions in regulating *E. coli* virulence. Although not all AHL signals in nature environment could bind to *E. coli* SdiA and regulate its pathogenicity, function of QS-1 signals from *P. aeruginosa* YZ1 and *A. hydrophila* YZ2 upon *E. coli* virulence have been confirmed, which strongly support that hypothesis. The data described here represent a step forward in the successful isolation and characterization of AHL-synthase positive bacteria from cattle rumen and pig intestines, and offer us a new vision to consider the multi-relationship between *E. coli* and gastro-intestinal tract in infection process through quorum sensing.

## Author contributions

GZ, PH, and YY conceived and designed the experiments. MZ isolated and analyzed YZ1. YY isolated and analyzed YZ2. YY and HC wrote this paper.

### Conflict of interest statement

The authors declare that the research was conducted in the absence of any commercial or financial relationships that could be construed as a potential conflict of interest.
